# Using Deep Neural Networks for Human Fall Detection Based on Pose Estimation

**DOI:** 10.3390/s22124544

**Published:** 2022-06-16

**Authors:** Mohammadamin Salimi, José J. M. Machado, João Manuel R. S. Tavares

**Affiliations:** 1 Faculdade de Engenharia, Universidade do Porto, Rua Dr. Roberto Frias, s/n, 4200-465 Porto, Portugal; up202009606@edu.fc.up.pt; 2 Departamento de Engenharia Mecânica, Faculdade de Engenharia, Universidade do Porto, Rua Dr. Roberto Frias, s/n, 4200-465 Porto, Portugal; jjmm@fe.up.pt

**Keywords:** image analysis, computer vision, machine learning, deep learning

## Abstract

Requests for caring for and monitoring the health and safety of older adults are increasing nowadays and form a topic of great social interest. One of the issues that lead to serious concerns is human falls, especially among aged people. Computer vision techniques can be used to identify fall events, and Deep Learning methods can detect them with optimum accuracy. Such imaging-based solutions are a good alternative to body-worn solutions. This article proposes a novel human fall detection solution based on the Fast Pose Estimation method. The solution uses Time-Distributed Convolutional Long Short-Term Memory (TD-CNN-LSTM) and 1Dimentional Convolutional Neural Network (1D-CNN) models, to classify the data extracted from image frames, and achieved high accuracies: 98 and 97% for the 1D-CNN and TD-CNN-LSTM models, respectively. Therefore, by applying the Fast Pose Estimation method, which has not been used before for this purpose, the proposed solution is an effective contribution to accurate human fall detection, which can be deployed in edge devices due to its low computational and memory demands.

## 1. Introduction

The hospital admission rate due to falls of people over 60 years old in Canada, Australia, and the United Kingdom range from 1.6 to 3.0 per 10,000 population [1,2]. According to [2], between 28 to 34% of old adults had experienced at least one fall every year. Moreover, falling is the second most significant reason for accidental death for more than 87% of adults in old age [3]. However, the detection of human falls in images is very complex as such events can be easily mistaken for other activities. For instance, actions such as sitting down, bending, or lying down on a bed or sofa, can assume similar poses to falls.

According to [1], fall detection may be a sub-task of general human activity estimation, presenting distinguishable characteristics to general motion detection. Usually, there is a quick change in height and width of the body after a fall, followed by an inactivity period on the ground, which can be identified by the sleeping pose or a lack of head movement [4].

The usual approaches for fall detection can be classified into two distinct groups: vision and non-vision based. A non-vision approach relies on wearable devices that use internal sensors, like accelerometers, for fall detection [2]. These approaches have their drawbacks, such as difficulties of usability, acceptance from users to wear them most of the time, or power efficiency of the devices; additionally, such sensors are prone to creating high false warnings being therefore not reliable [5]. Therefore, less intrusive and demanding vision-based approaches have been proposed [6].

Usually, in vision-based approaches, the person’s motion is obtained from image frames and then analyzed to detect events like lying on the floor, sitting, or standing. The features used in such approaches are shape, posture, pixel information, distance, head position, and orienting angle, amongst others [4].

Numerous approaches that have been proposed for fall detection lead to results with minor errors, especially using computer vision-based techniques. Lin et al. [7] proposed a computer vision algorithm by combining shape-based fall characterization and a supervised-learning classifier, in order to identify and distinguish falls from other daily actions. The algorithm used the Curvature Scale Space (CSS) feature of human silhouettes extracted from each image frame to represent the imaged action and was tested on an action dataset acquired using the Kinect depth camera. The used dataset included six types of actions: falling, bending, sitting, squatting, walking, and lying, performed by ten subjects. The authors stated that one of the challenges for computer vision-based methods for accurate fall detection is the lack of a large fall dataset, and some difficult intrinsic factors such as image acquisitions with different view angles, illumination conditions, and clothes. Jiao et al. [8] addressed the mentioned problems by using an improved Recurrent Neural Network (RNN) with the Long Short-Term Memory (LSTM) architecture in order to model the temporal dynamics of the 2D pose information of a fallen person, which led to an improvement over the existing models.

Human pose estimation has been the main topic in computer vision, which has been extensively implemented for action recognition and human-computer interaction [6]. Usually, it requires an efficient process to detect and locate body key points, such as wrists, head, elbows, and knees, to name a few, from the input image frames. By implying deep Convolutional Neural Networks (CNNs), significant improvements have been achieved in this area [7,8]. However, concerning fall detection, deep networks with complex models are time-consuming at inference time based, and demand high computing power and memory space. Therefore, the deployment of the top-performing deep learning networks on embedded systems is frequently not suitable, which motivated this study to develop an agile fall detection method by using fast human pose estimation that presents lightweight computation cost and high accuracy [9].

The used bi-dimensional (2D) posture model was influenced by the Real-time Human Pose Estimation in TensorFlow.js. Then, to recover the third dimension from the 2D pose, a deep feedforward neural network was trained on the 2D joints, which were obtained using a motion capture system, in order to estimate their depth in the coordinate system of the scene [9]. Every second, the detection model ran in the background to tackle incoming persons, while a simple bounding box tracking was employed to monitor every person in the scene. The proposed model could detect multiple persons in outdoor and indoor scenarios. The Fast Pose model was implemented using Python 3.6 software and the TensorFlow, PyTorch, OpenCV, and Matplotlib toolkits [10].

**Figure 1:**
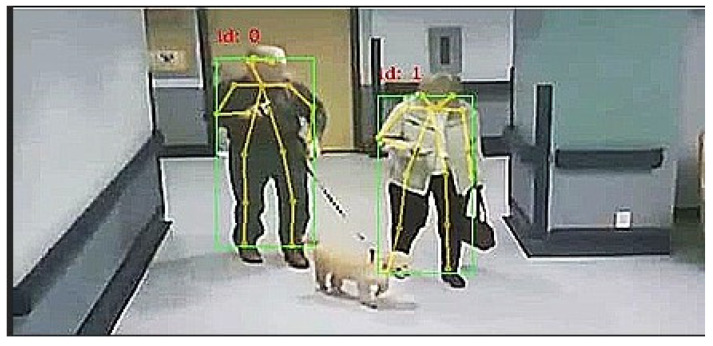
Examples of the detection of key points by the Fast Pose model.

The primary goal of this study was to assess the performance of the Fast Pose model to classify fall and non-fall image frames, i.e., videos, by using lightweight deep learning networks. Fast Pose is a short and open-source library that can perform 2D/3D pose estimation in real-time on both Central Processing Units (CPUs) and Graphics Processing Units (GPUs) [8]. Initially, it detects a skeleton, which consists of key points and connections between them, to identify human poses for persons in image frames. The poses may contain 18 key points found in the shoulders, neck, nose, elbows, wrists, hips, knees, and ankles, Figure 1. The detection of the size and location of the human body is based on a top-down based approach. Then, the input image is cropped around the regions of interest and used for pose inference [8].

Most of the vision-based fall detection approaches rely on the use of background subtraction to firstly distinguish the objects of interest in the images from the scene, i.e., image, background, and then detect the fall events [11]. In Ref. [9], an approach that used a ceiling-mounted camera designed to detect background deduction full-body images, and categorized them with the vectorized silhouette of their shape was proposed. However, one of the drawbacks of the proposed approach is the lack of success in detecting “tucked” falls, mainly in public areas [9].

In early human pose estimation methods, it was common to develop the image structure model using hand-crafted features [1,11]. However, there was an important challenge for the traditional pose estimation methods, since they could lead to inaccurate detections when some parts of the body were occluded. With the emergence of deep convolutional neural networks for human pose estimation, which is reviewed, for example, in [12,13], the models can learn how to predict human pose on large-scale datasets with intensive human joint annotations.

According to Luo et al. [14], traditional methods adopt different hand-crafted feature extraction methods for body parts. These techniques often have poor performance and extra computational costs, but by proposing the Deep Pose method based on deep learning approaches, the performance has been effectively improved. In addition, multi-scale fusion can improve the accuracy of human pose estimation; for example, by using the HRnet and HIgherHRnet models that accept input from parallel subnetworks [14]. By repeatedly performing multi-scale fusions among these parallel multi-resolution subnetworks, the HRNet can obtain rich high-to-low resolution representations, leading to enhanced high-resolution representations [10].

Most of the works in this area are mainly focused on how to design a top-performing pose estimation method by adopting complex architectures or expensive computer models, originating the need for new solutions with lower computational and memory demands that can be deployed in edge devices. Therefore, the development of a lightweight and optimized solution based on a novel pose estimation model [15] represents an advantage for this field.

The Fast Pose Distillation (FPD) model can be used to more effectively train extremely small human pose convolution neural networks [16]. It consists of knowledge extraction based on object image classification using deep learning models. In short pose estimation, the pose knowledge distillation objective is to transfer the latent knowledge from a pre-trained larger learning model to a tiny target pose model to be deployed in test time [16]. In Ref. [17], real-time in multi-person 2D pose estimation was used to retrieve the location of 25 joint points of the human body, and detect the human movement based on their locations.

Traditional articulated human pose estimation has been formulated as a structured prediction task requiring an inference step that combines local observations of body joints with spatial constraints [18]. The purpose of the inference process is to convert observations from local part detectors into coherent body configuration estimations. Powerful body component detectors have gradually surpassed these models, which have been strengthened by the development of robust image representations based on convolutional networks [19]. Recent research attempted to add convolutional detectors into part-based models or to create stronger detectors by integrating the detectors’ output with location-based characteristics [20]. For example, in [11], a fall detector system uses a CNN-based human pose estimation combined with stereo data to reconstruct human pose in 3D and estimate the ground plane in 3D. Taking into account different scenarios covering most activities of people living at home, an extensive evaluation demonstrated high accuracy (91%) without missed classifications. The main contribution of this research was to implement the fast pose method as a lightweight network and tested it using an augmented image dataset. The proposed solution achieved the highest accuracy among similar state-of-the-art computer vision-based methods for fall detection and demands low computational resources, which is an advantage for being deployed in edge devices, for example.

## 2. Model Architecture and Model Training

The main purpose of this study was to use one type of light human pose estimation method, mainly the Fast Pose estimation method [15], for human fall detection. In this method, the *first* step is to build a highly cost-effective human pose estimation model, which requires the development of a compact backbone such as the Hourglass network [21]. To more effectively train a small target learning network, the principle of knowledge distillation in the pose estimation context was adopted. The *second* step of the method requires the pre-training of a strong pose model trainer, such as the state-of-the-art Hourglass network or another existing alternative. The trainer model is then used to provide extra supervision guidance in the pose knowledge distillation procedure via the proposed limitation loss function. Finally, the test step is conducted, aiming to achieve a small target pose model that provides a fast and cost-effective deployment [15].

**Figure 2:**
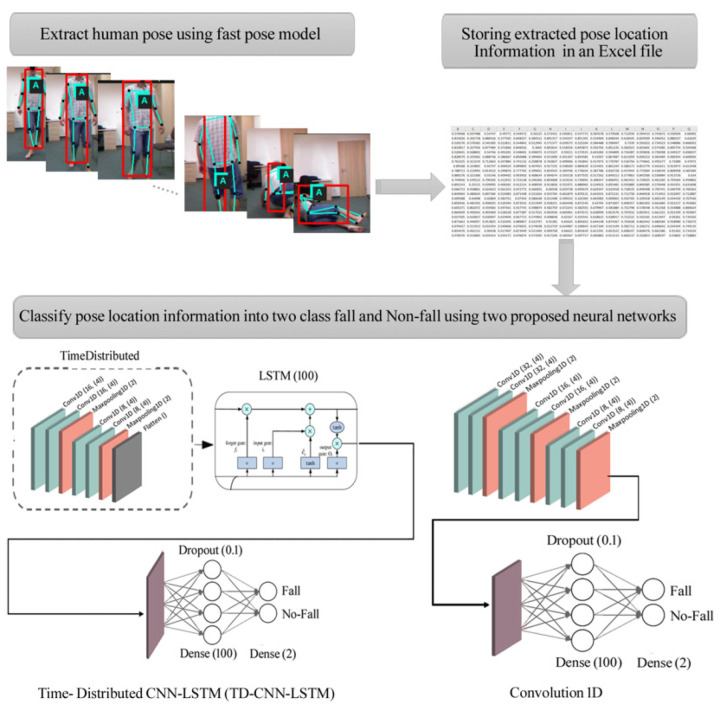
Flowchart of the proposed method for fall detection from image frames.

Figure 2 shows a flowchart of the proposed solution for fall detection in image frames. It consists of four steps: (1) recording fall and non-fall videos, (2) human pose extraction using the Fast Pose method from the videos, (3) storing the coordinates of extracted pose positions, and (4) implementing 1D-CNN and Time-Distributed CNN-LSTM (TD-CNN-LSTM) models to classify fall and non-fall events using the stored coordinates.

The body’s position during falls or Daily Life Activities can be used as a characteristic for classifying them in image frames. A sequence of images is used to monitor the changes in the human body pose providing, therefore, a significant clue to identify falls from other daily activities [20,22]. Tracking is always challenging when considerable motion is involved. The idea is that body pose can be applied to every image frame and considered in the tracking strategy.

The UR Fall Detection Dataset (URFD) used in this study contains 70 video sequences, including 30 falls and 40 daily living activities. Since the original dataset is considerably small for training and testing the developed models, techniques of data augmentation (DA) [23] were used in order to improve the models’ generalization and performance. Thus, the use of the data augmentation techniques, mainly rotation, horizontal flip, brightness, and gamma correction, on the original videos allowed us to obtain a total of 560 videos: 240 of falls and 320 of daily living activities.

One of the novelties of this work is related to the analysis of the location of key points of the human body, which are extracted by the fast pose estimation method [15]. Therefore, the use of the fast pose estimation method led to the dataset used to train the proposed models. For example, using the fast pose estimation method, a total of 6110 data samples, i.e., the position of body key points, were extracted from just a non-fall video. Therefore, the position of key points from all image frames of all experimental videos provided a huge dataset to be used to train the proposed deep learning models in order to detect if a person is lying on the ground. The comprised living activities included everyday events, such as walking, lying down, bending, sitting down, and crouching down. On the other hand, the image frames to be evaluated were recorded in standard rooms, including a living room, office, and classroom, having just a tiny amount of furniture in the acquired scene. Their duration ranges from 2 to 13 s, and the number of frames included per second is 30.

A fall sequence shows the rapid and sudden change of pose location compared with daily living activities. Therefore, this change can be used to detect a fall based on the tracking of the human pose. In this step, by running the fast pose model on the input image frames, the location information of 18 poses is extracted in each frame and automatically stored [16]. Then, this data is fed separately to both TD-CNN-LSTM and 1D-CNN models to classify it into two classes: fall and non-fall.

LSTM models can learn sequence dependence information and accurately predict sequential data. This deep learning network has an internal state that uses context information learned from previous time steps to influence the prediction at the current time [22]. Additionally, a CNN can automatically extract features and provide informative time series representations. It consists of a highly noise-robust model that can extract valuable information and in-depth features independent of time. Using joint location information and short pose estimation, it is possible to analyze the change rate of poses in a sequence of image frames, with this being information used by the LSTM and one-dimensional CNN classifiers to detect falls and non-falls.

## 3. Convolution Neural Network

Deep Learning neural networks have been used to successfully extract significant features for time series classification. Particularly, a CNN is an integrated framework that simultaneously can perform feature extraction and classification tasks [24], and it includes three main layers: convolution, pooling, and fully connected layers. Each layer operates in different ways to conclusive learning. This network has been applied alone or together with other networks in different architectures for data classification. The following steps allow classifying pose location information based on a CNN model.

### 3.1. Feature Extraction

**Figure 3:**
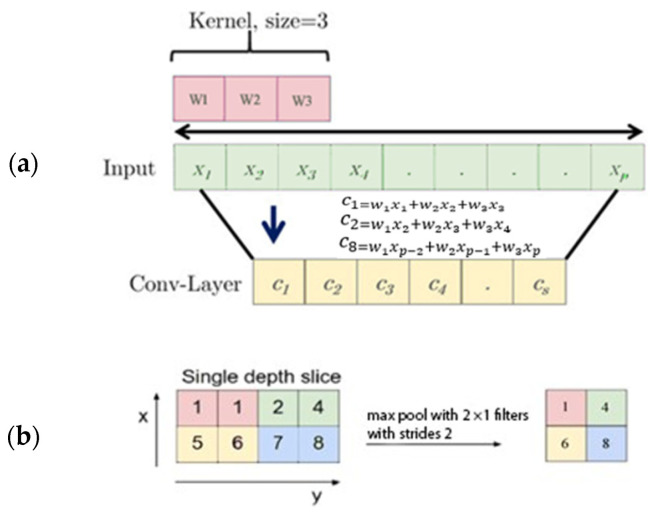
A 1D-CNN operator with a kernel of size 3 (**a**), and a Max pooling with a single pooled feature (**b**).

A CNN uses convolution and pooling layers to monitor and evaluate features in the first step; Figure 3 illustrates how the filter kernel extracts valuable features. Then, the max-pooling layer decreases the size of the extracted features. It can tackle the overfitting problem in the neural network training step. It also evaluates the zone’s maximum from the extracted feature map created by the convolution layer.

### 3.2. Classification

**Figure 4:**
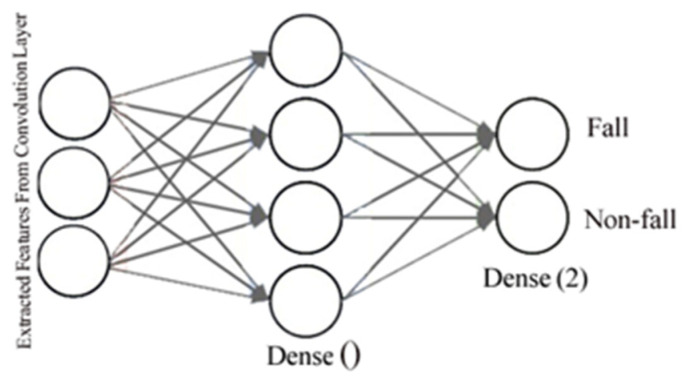
Example of a Fully Connected layer.

An activation function and a dropout layer can cause non-linearity and decreased overfitting. As shown in Figure 4, a fully connected layer can be used to categorize the features into two classes; here, fall and non-fall classes.

**Figure 5:**
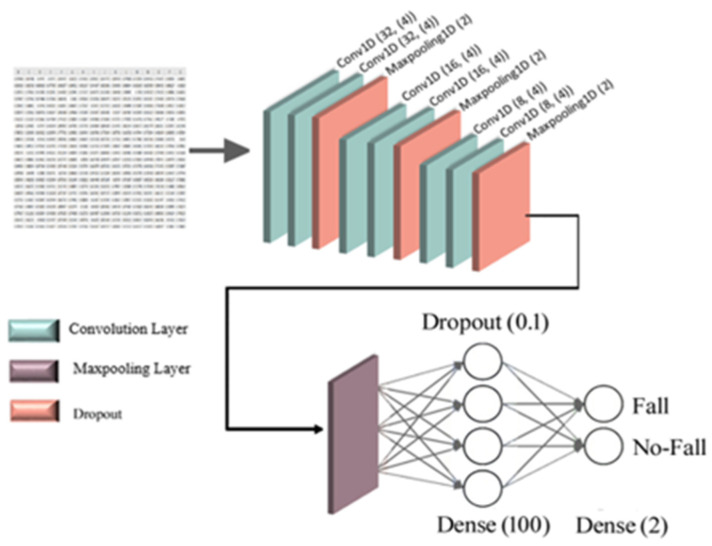
Schematic representation of the proposed 1D-CNN architecture.

Figure 5 presents the architecture of the one-dimensional CNN used in this study. Of the used 70 videos, 80% (56 videos) were used as training data and 20% (14 videos) as testing data. The used model includes two convolution layers of 32 filter length, two convolution layers of 16 filter length, and two convolution layers of 8 filter length. Commonly, in a convolution neural network, the convolution layer is followed by a pooling layer with one or several fully connected layers. However, applying a pooling layer after each convolution layer is not necessary.

It is possible to realize from Figure 5 that the used model consists of 6 convolution layers and three max-pooling layers. The last max-pooling layer output is a 2D array, converted into a 1D array and sent to the fully connected layers using a flattened layer. To control the output size of each convolution layer, a type of padding should be used. Here, the same padding for the two proposed neural networks was used. The activation function was the Relu function, and in the last layer the SoftMax activation function was used, and then, 1024 fully connected layers and two fully connected layers were used to classify the input data into the two classes of interest. The Adam function was used as an optimization function because the results were significantly better than those of other optimization functions, such as SGD and Adadelta. The learning rate (LR) parameter was tested with values of 0.0001 and 0.00001, being the best result obtained using the latter. The network training was over 300 epochs, being the data passed to the network in batches of 8 sizes (batch-size). The time of all epoch training was 1 min, which means that the duration of each epoch was 0.2 s.

The number of the used parameters depends on the output size of the last convolution layer, the number of filters, and the number of hidden layers that are fully connected. According to Table 1, the output shape of the first convolution layer of the used 1D-CNN was (6110, 32), where 6110 refers to the input data size, and 32 to the filter size of the first layer of the model. In the first stage, the number of parameters was 160. The output shape was the same as the first layer for the next convolution layer, and the number of parameters was (1024 × 4) + 32 = 4128, where 1024 represents the number of parameters in the network’s hidden layer, 4 the convolution layer’s window size, and 32 the convolution layer’s filter size (Conv1D 32, (4)).

Based on the architecture of the used 1D-CNN, there was a max-pooling layer with stride 2 after two convolution layers, with the output of the convolution layer divided by two. Therefore, the output shape of the first max-pooling layer was 6110/2 = 3055, and the number of parameters for max-pooling layers was equal to 0 (zero). The next layer was a convolution layer with a filter size of 16. The output shape was (3055, 16) for this layer, and the number of parameters was (512 × 4) + 16 = 2064, where 512 denotes the number of parameters in the network’s hidden layer, 4 the window size of the convolution layer, and 16 the filter size of the convolution layer (Conv1D 16, (4)). Here, the number of hidden layer parameters was divided by two after each convolution layer. For the next convolution layer, the output shape was (3055, 16), and the number of parameters was (256 × 4) + 16 = 1040, where 256 refers to the number of the parameters in the hidden layer, 4 to the window size of the convolution layer, and 16 to the filter size of convolution layer (Conv1D 16, (4)). Then, the output shape for the max-pooling layer was 3055/2 = 1527.5~1528 with a parameters number of 0 (zero). The output shape was (1528, 8) for the next convolution layer, and the number of parameters was (128 × 4) + 8 = 520. For the last convolution layer, the output shape was the same as of the last convolution layer with a parameters number of (64 × 4) + 8 = 264, where 64 refers to the parameters number of the hidden layer, 4 to the window size of the convolution layer, and 8 to the filter size of convolution layer (Conv1D 8, (4)). The number of extracted features from the last max-pooling layer of networks was 1528/2 = 764, which was used as input for the flattening layer. So, the output of the flattening layer was 764 × 8 = 6112. In this stage, the output of the flatting layer multiplied by dense layer size was (6112 × 1024 = 6258,688) and a bias of 1024 was added to the result: 6,258,688 + 1024 = 6,259,712. During the training step, some number of layer outputs were randomly ignored or dropped out by the dropout layer. The output shape of the dropout layer was 1204, so the parameters number of the last dense layer with size 2 was (1024 × 2) + 2 (biases) =2050. Finally, by adding up all the values of the parameters in Table 1, it is possible to calculate the total number of parameters associated with the network training, in this case: 6,269,938.

### 3.3. CNN-LSTM

**Figure 6:**
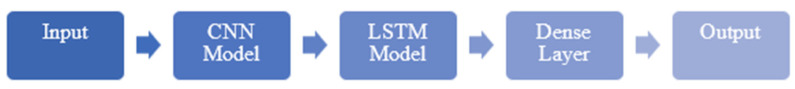
The primary process of the CNN-LSTM model.

LSTM is a robust neural network able to learn how to bridge minimal time lags over 1000 discrete time steps by enforcing constant error flow in special units. Multiplicative gate units identify and grasp the potential to open and close access to the constant error flow. Besides this, LSTM is local in space and time and improves the RNN architecture [25], and its main strength lies in dealing with sequential data such as in time series [26]. A CNN-LSTM consists of convolutional layers to extract input data features, combined with LSTMs to support sequence prediction. CNN-LSTM was developed for time series prediction problems and for producing textual descriptions from sequences of images, i.e., videos, Figure 6. This model divides the primary data sequence into sub-sequences as blocks, extracts features from each block, and then interprets each block’s extracted features. Although LSTMs are robust neural networks, it is hard to use and configure them. Adding a complex Time-Distributed Layer as a layer wrapper allows applying a layer to every temporal slice of input [27].

The time-distributed structure serves to learn the long-term and short-term features of the time series. Consequently, it makes perfect use of extracted information on different time scales. The Time-Distributed method converts the raw time-series input into a shorter sequence, making learning long temporal dependencies easier. Time-Distributed CNN-LSTM (TD-CNN-LSTM) is a composite end-to-end framework that uses time-distributed spatiotemporal feature learning, and so a deployment method of basic CNN-LSTM [26].

**Figure 7:**
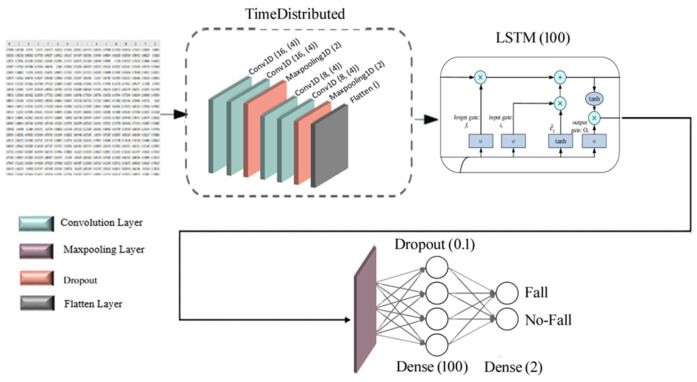
The architecture of the proposed TD-CNN-LSTM classification model.

For this network, the output shape of the first convolution layer was (6110, 16), where 6110 is the input data size, 16 refers to the window size of the convolution layer, and the parameters number of the first convolution layer was 32. The output shape was equal to the first layer for the second convolution layer, and the number of parameters was (64 × 4) + 16 = 272, where 64 is the number of the hidden layer, 4 refers to the window size of the convolution layer, and 16 to the filter size of the convolution layer. There was a max-pooling layer with stride 2 after two convolution layers, dividing the convolution layer’s output by two. Therefore, the output shape of the first max-pooling layer was 6110/2 = 3055, and the number of parameters for max-pooling layers was equal to 0 (zero). The next layer was a convolution layer with an output shape of (3055, 8) and a parameters number of (32 × 4) + 8 = 136, where the number of the hidden layer, the convolution layer’s window size, and the convolution layer’s filter size were: 32, 4, and 8, respectively. The output shape of the next layer was equal to the one of the last layer, and the number of the parameter was (16 × 4) + 8 = 72, where the number of the hidden layers, the window size of the convolution layer, and filter size of the convolution layer were: 16, 4, and 8, respectively. The next max-pooling layer had an output shape of 3055/2 = 1527.5~1527 with a parameters number of 0 (zero). After the last max-pooling layer, a flatten layer converts the two-dimensional output to a one-dimensional output. So, the output of the flatten layer was equal to 1527 × 8 = 12,216. According to Figure 7, the output of the flatten layer was the input for an LSTM layer with an output shape of 100 and a parameters number equal to 4,926,800. There is no exact way to compute the number of parameters during a LSTM training process. A dense layer with 1024 is used after the dropout layer which was shaped as 100. For this layer, the number of parameters was (100 × 1024) + 1024 (biases) = 103,424. The output shape of the dropout layer was 1204, so the parameters number of the last dense layer with size 2 was (1024 × 2) + 2 (biases) = 2050. Adding up all the values of the Number of Parameters column of Table 2 allows us to determine the total number of parameters associated with the training of this model: 5,032,786.

## 4. Experimental Results

This study used the Fast Pose Model for human pose detection and to classify detected pose sequences into fall and non-fall classes. For this purpose, two deep neural network models were implemented and evaluated in terms of accuracy, precision, recall, and F1-score criteria. Accuracy refers to the proximity of a measured value to a standard or actual value [19]:

Accuracy = (TP + TN)/(TP + TN + FP + FN)(1)

where TP refers to true positive, TN to true negative, FP to false positive, and FN to false-negative cases.

Precision is a metric that assesses the number of correct predictions made, calculated as the proportion of correctly predicted positive samples divided by the number of positive samples forecasted. Both precision and recall are metrics that can be combined to evaluate the performance of classification or information retrieval systems. However, some feature extraction strategies are complex, and usual machine learning classifiers have particular demands as to the used datasets, due to their limited ability of generalization [28]. Precision is the fraction of relevant instances among all retrieved instances, and Recall, sometimes referred to as “sensitivity”, is the fraction of retrieved occurrences among all relevant instances:

Precision = TP/(TP + FP)(2)

Recall = TP/(TP + FN)(3)

A perfect classifier has both precision and Recall equal to 100%. It is frequently possible to calibrate a classifier and improve its precision at the expense of the recall, or contrariwise [15].

Precision and recall are sometimes combined into the F1-score if a single numerical measurement of a system’s performance is required [13]:

F1-Score = (2 × (Recall × Precision))/((Recall + Precision))(4)

Table 3 summarizes the results obtained by the 1D-CNN and TD-CNN-LSTM proposed classifier models. The training accuracy of the implemented 1D-CNN was 99.13%, and its validation accuracy was equal to 99.31%. The training and validation accuracies of the implemented TD-CNN-LSTM were 98.90 and 99.08%, respectively.

**Figure 8:**
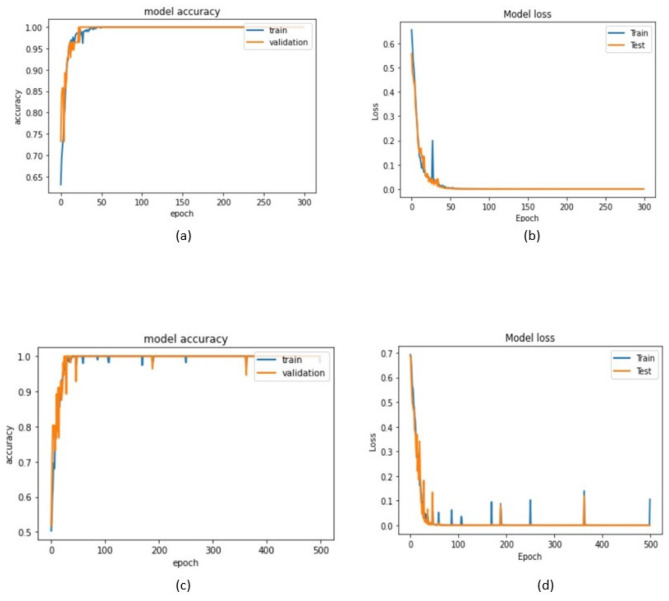
Training and validation analysis of overall epochs for the developed 1D-CNN model: (**a**) Accuracy analysis of Training and Testing, and (**b**) Loss analysis of Training and Testing; and for the developed TD-CNN-LSTM model: (**c**) Accuracy analysis of Training and Testing, and (**d**) Loss analysis of Training and Testing.

Figure 8 presents the training and validation accuracies and loss analysis of the developed models concerning the number of epochs, and, from them, it can be realized that, for both models, the training loss was in the minimum range, and the accuracy was higher than 97%.

Training a learning model means learning good values for all the weights and biases from labelled examples [25]. A loss is a number indicating how wrong the model’s prediction is on a single example. If the model’s prediction is flawless, the loss is zero; if not, the loss is more significant. Hence, training a model means finding a set of weights and biases that have low loss, on average, across all examples [23]. The common problem in training learning models is related to the overfitting that can occur during the process, which was observed in the primary model of this network but, by changing the size of max-pooling and the second Dense layer of the network, the problem was solved. According to Figure 8, the model’s accuracy rate increased, and the loss decreased by running more training epochs.

Table 4 summarizes the precision, recall, and F1-score for the two classes under study obtained by the implemented 1D-CNN and TD-CNN-LSTM models.

According to the results in Table 4, both proposed models achieved high evaluation metrics, confirming their reliability to predict fall and non-fall events in image frames.

**Figure 9:**
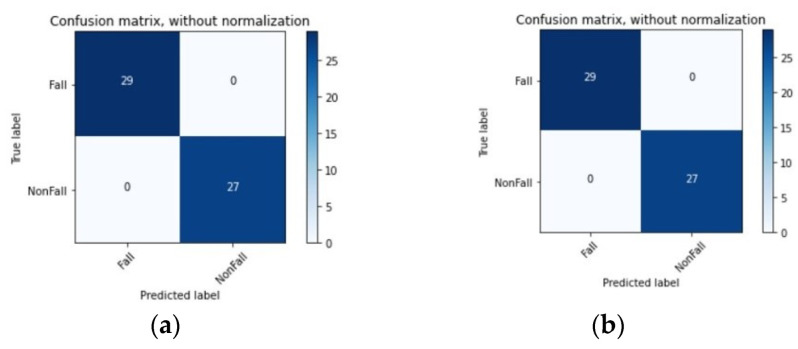
Confusion Matrix obtained by the proposed 1D CNN (**a**) and TD-CNN-LSTM (**b**) models on the testing data.

In the research field of artificial intelligence, a confusion matrix is often used to visualize an algorithm’s performance. In this matrix, each column represents the predicted value of instances, and each row represents the actual, i.e., true, value of instances. Figure 9 shows the confusion matrices built for the two proposed models to visualize whether they predicted correctly or not. In these matrices, the classification of 29 and 27 samples obtained by the implemented 1D-CNN and TD-LSTM CNN models are represented, respectively, being possible to confirm their high accuracy in predicting the two classes under study.

**Figure 10:**
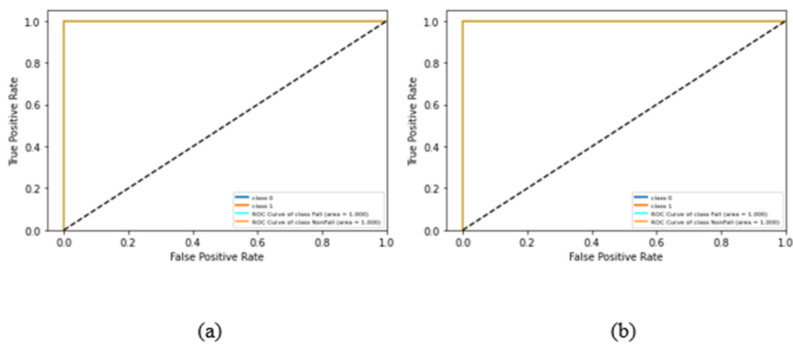
ROC curves built for the implemented TD-CNN-LSTM (**a**) and 1D-CNN (**b**) models.

Receiver Operating Characteristics (ROC) curves help to evaluate and benchmark classifiers, allowing the visualization of their performance. ROC plots are frequently used in clinical decision-making and have recently become essential in machine learning and data mining. The ROC plot is created by plotting the True Positive Rate (TPR) versus the False Positive Rate (FPR) in different threshold settings. TPR denotes the number of items correctly classified as positive by the classifier. On the other hand, FPR is the number of items wrongly classified as positive by the classifier. Lowering the FPR, thus maximizing the TPR, allows for achieving an optimal status. Therefore, the optimal point (TPR = 1 and FPR = 0) is in the upper-left corner of the ROC curve. Figure 10a shows the ROC curve built for the implemented TD-CNN-LSTM model, and Figure 10b shows the ROC curve built for the implemented 1D-CNN model, according to the 0 (fall) and 1 (non-fall) classes under study. The calculation of the area under the ROC curve, also known as the area under the curve (AUC), has the maximum, which is the best value, of 1.00. Regarding the ROC curves in Figure 10, the AUC of the TD-CNN-LSTM model was optimal (area = 1.00), and the AUC of the 1D-CNN model was in a similar range (area = 1.00), which confirms once more the very good performance achieved by the proposed classifier models.

Several authors used pose estimation to achieve accurate fall detection by machine learning and deep learning methods in image frames. Table 5 allows a comparison of the results obtained by similar research works. This comparison shows that the proposed 1D-CNN model outperformed the proposed TD-CNN-LSTM model by 1% in terms of accuracy.

From Table 5, it is also possible to compare the results of the proposed models against the ones obtained by other studies also on the URFD dataset. In Ref. [27] by using the Open-pose estimation method, key points of the skeleton were detected, and a LSTM model provided an accuracy of 92% for fall detection. According to [27], a recurrent neural network with a LSTM architecture that models the temporal dynamics of the 2D pose information of a fallen person was developed. Human 2D pose information, which has proven to be effective in analyzing fall patterns, as it ignores people’s body appearance and environmental information while capturing accurate motion information, made the proposed model simpler and faster. In Ref. [29], the combination of Exponentially Weighted Moving Average, a monitoring scheme, and an SVM classifier was proposed to classify input data into fall and non-fall classes. The solution achieved an accuracy of 96% and a sensitivity of 100%. The disadvantage of this solution relies on failure to differentiate actual falls from some fall-like gestures, so, to overcome this barrier, a classification stage based on a support vector machine was applied to detected sequences. Another method based on the pose estimation method was suggested in [30] by adopting an LSTM model to detect falls from continuous human activity, which achieved a 97.38% accuracy. Núñez-Marcos et al. [31] achieved an accuracy of 95% by proposing a network to solely analyze the situation of the whole body in image sequences. The work of Hasan et al. [24], which is focused on human 2D pose information, has shown to be effective in analyzing fall patterns by achieving 99% of sensitivity. This model had one of the most promising performances among the state-of-the-art reviewed works, but it was still 1% lower than the proposed TD-LSTM-CNN and 1D-CNN models, which achieved 100% of sensitivity.

According to the experimental results, the proposed models for human fall detection achieved very promising accuracies when compared to the reviewed related state-of-the-art methods. There is a lack of contributions related to optimized lightweight human pose methods for human fall detection that can be applied efficiently with low computational demands. In terms of evaluation metrics, the TD-LSTM-CNN and 1D-CNN models achieved the highest values, which are superior to those of all reviewed related works. Therefore, this research represents an important contribution to this field, since the Fast pose estimation method was proved to be highly competent in fall detection demanding low computational resources by using a lightweight human pose-based approach.

## 5. Conclusions

This article proposed a fall detection solution based on the Fast-Pose estimation method, which is based on the extraction from the input image frames of the human skeleton, the detection of the body’s critical points, and their further classification using deep learning models. An augmented version of the URFD dataset, was obtained by using the rotation, brightness, horizontal flip, and gamma correction augmentation techniques on the original URFD videos, which led to a total of 560 videos, including 240 videos of falls and 320 videos of daily life activities, was used to assess the developed models.

At first, the location information of human poses in each image frame is extracted by using the Fast-Pose estimation method and stored. The classification of extracted data into fall and non-fall classes is achieved by applying 1D-CNN and CNN-LSTM models. Regarding the evaluation results achieved by both proposed models, the accuracy obtained by the 1D-CNN model was equal to 99.13%, which is higher than those obtained by similar state-of-the-art methods. This achievement represents an advantage of the proposed model. The proposed models were also assessed using other evaluation criteria such as recall, precision, and F1-score. The highest precision, recall, and F1 score (of 100%) values were obtained by both proposed models. These findings also confirm that the proposed solution, which is based on the fast pose method that has not been used before for this purpose, is an effective way to accurately detect human falls in image frames, requiring very low computational resources being, therefore, very interesting to be deployed in edge devices.

To evaluate the generalization of the proposed models, other datasets that include different views of fallen people, and not just the front view, and the use of other augmentation techniques, mainly based on Generative Adversarial Networks (GANs), are suggested for future work.

## Figures and Tables

**Table 1:** Parameters used in the implemented 1D-CNN for the 2 classes problem under study.

Layer (Type)	Output Shape	N. of Parameters
Convld_8 (Convld1D)	(None, 6110, 32)	160
Convld_9 (Convld1D)	(None, 6110, 32)	4128
max_pooling1d_4 (Maxpooling1)	(None, 3055, 32)	0
Convld_10 (Convld1D)	(None, 3055, 16)	2064
Convld_11 (Convld1D)	(None, 3055, 16)	1040
max_pooling1d_5 (Maxpooling1d)	(None, 1528, 16)	0
Convld_12 (Convld1D)	(None, 1528, 8)	520
Convld_13 (Convld1D)	(None, 1528, 8)	264
max_pooling1d_6 (Maxpooling1)	(None, 764, 8)	0
flatten_2 (Flatten)	(None, 1024)	0
dense_4 (Dense)	(None, 1024)	6,259,712
dropout_2 (Dropout)	(None, 1024)	0
dense_5 (Dense)	(None, 2)	2050

Total number of parameters: 6,269,938; Number of trainable parameters: 6,269,938; Number of non-trainable parameters: 0.

**Table 2:** Parameters used in the implemented TD-CNN-LSTM for the 2 classes classification studied problem.

Layer (Type)	Output Shape	N. of Parameters
**t**ime_distributed_7	(None, None, 6110, 16)	32
**t**ime_distributed_8	(None, None, 6110, 16)	272
**t**ime_distributed_9	(None, None, 3055, 16)	0
**t**ime_distributed_10	(None, None, 3055, 8)	136
**t**ime_distributed_11	(None, None, 3055, 8)	72
**t**ime_distributed_12	(None, None, 1527, 8)	0
**t**ime_distributed_13	(None, None, 12216)	0
lstm_1(LSTM)	(None, 100)	4,926,800
dropout_1(Dropout)	(None, 100)	0
dense_2 (Dense)	(None, 1024)	103,424
dense_3 (Dense)	(None, 2)	2050

Total number of parameters: 5,032,786; Number of trainable parameters: 5,032,786; Number of non-trainable parameters: 0.

**Table 3:** Results of the proposed models for the two classes classification problem under study.

Model	Training Accuracy	Validation Accuracy	Training Loss	Validation Loss
1D-CNN	0.9913	0.9931	0.0196	0.0181
TD-CNN-LSTM	0.9890	0.9909	0.0221	0.0196

**Table 4:** Precision, recall, and F1-score values were obtained by the proposed deep neural network models.

Fall				Non-Fall		
Model	Precision	Recall	F1-Score	Precision	Recall	F1-Score
1D-CNN	1.0	1.0	1.0	1.0	1.0	1.0
TD-CNN-LSTM	1.0	1.0	1.0	1.0	1.0	1.0

**Table 5:** Classification results obtained by the proposed and other published related models based on pose estimation for fall detection in image frames.

Reference	Method	Accuracy	Precision	Sensitivity
Lin C et al. [27]	Open Pose-LSTM Model	92%	-	-
Hasan M et al. [24]	2DPose estimation	-	-	99%
Nunez A et al. [31]	2D Pose estimation	95%	-	-
Harrou F et al. [29]	SVM	96.66%	-	100%
Jeong S et al. [30]	LSTM	97.38%	-	-
Proposed 1D-CNN	1D-CNN	99.13%	100%	100%
Proposed TD-CNN-LSTM	TD_CNN-LSTM	98.90%	100%	100%

## Data Availability

Not applicable.
